# Risk of hospital admission for patients with SARS-CoV-2 variant B.1.1.7: cohort analysis

**DOI:** 10.1136/bmj.n1412

**Published:** 2021-06-15

**Authors:** Tommy Nyberg, Katherine A Twohig, Ross J Harris, Shaun R Seaman, Joe Flannagan, Hester Allen, Andre Charlett, Daniela De Angelis, Gavin Dabrera, Anne M Presanis

**Affiliations:** 1 MRC Biostatistics Unit, University of Cambridge, Cambridge, UK; 2 COVID-19 National Epidemiology Cell, Public Health England, London, UK; 3 National Infection Service, Public Health England, London, UK

## Abstract

**Objective:**

To evaluate the relation between diagnosis of covid-19 with SARS-CoV-2 variant B.1.1.7 (also known as variant of concern 202012/01) and the risk of hospital admission compared with diagnosis with wild-type SARS-CoV-2 variants.

**Design:**

Retrospective cohort analysis.

**Setting:**

Community based SARS-CoV-2 testing in England, individually linked with hospital admission data.

**Participants:**

839 278 patients with laboratory confirmed covid-19, of whom 36 233 had been admitted to hospital within 14 days, tested between 23 November 2020 and 31 January 2021 and analysed at a laboratory with an available TaqPath assay that enables assessment of S-gene target failure (SGTF), a proxy test for the B.1.1.7 variant. Patient data were stratified by age, sex, ethnicity, deprivation, region of residence, and date of positive test.

**Main outcome measures:**

Hospital admission between one and 14 days after the first positive SARS-CoV-2 test.

**Results:**

27 710 (4.7%) of 592 409 patients with SGTF variants and 8523 (3.5%) of 246 869 patients without SGTF variants had been admitted to hospital within one to 14 days. The stratum adjusted hazard ratio of hospital admission was 1.52 (95% confidence interval 1.47 to 1.57) for patients with covid-19 infected with SGTF variants, compared with those infected with non-SGTF variants. The effect was modified by age (P<0.001), with hazard ratios of 0.93-1.21 in patients younger than 20 years with versus without SGTF variants, 1.29 in those aged 20-29, and 1.45-1.65 in those aged ≥30 years. The adjusted absolute risk of hospital admission within 14 days was 4.7% (95% confidence interval 4.6% to 4.7%) for patients with SGTF variants and 3.5% (3.4% to 3.5%) for those with non-SGTF variants.

**Conclusions:**

The results suggest that the risk of hospital admission is higher for people infected with the B.1.1.7 variant compared with wild-type SARS-CoV-2, likely reflecting a more severe disease. The higher severity may be specific to adults older than 30 years.

## Introduction

Since its discovery in England in November 2020, the SARS-CoV-2 B.1.1.7 variant has been reported in 135 countries globally.^1^ The prevalence of B.1.1.7 increased rapidly in England, and it became the predominant SARS-CoV-2 lineage by mid-December,^2^ prompting the re-implementation of social and physical distancing measures to control infection rates. These measures included closures of schools, non-essential retail, and hospitality outlets and stay at home orders.^3^

Initial concerns around B.1.1.7 emerged from analyses that determined a higher transmissibility.^2^ ^4^ ^5^ On 18 December 2020, the variant was redesignated as a variant of concern (VOC-202012/01), and subsequent studies have found B.1.1.7 to be associated with higher mortality than other SARS-CoV-2 variants.^6^ ^7^ ^8^ ^9^ ^10^

The burden of covid-19 on hospital services is a key component for progress in controlling the pandemic, influencing decisions on national preventive measures. Although its prevalence has now declined in England, B.1.1.7 is the predominant lineage in several countries, and any potential increased likelihood of hospital admission with this variant will affect the national healthcare burden in those countries.

Initial assessments of hospital admissions were based on ecological analyses, looking at distribution of variant cases compared with the levels of healthcare demand at different geographies.^5^ ^11^ ^12^ More recently, a higher risk of admission to critical care has been reported for patients with covid-19 tested in the community.^9^ One study, based on whole genome sequencing, has reported on the risk of hospital admission by using individual level follow-up of patients with covid-19 due to B.1.1.7 compared with wild-type SARS-CoV-2.^13^ However, that study was limited by a moderate sample size owing to operational constraints of sequencing, leading to wide confidence intervals for the risk estimates. Hospital admissions linked to individual variant cases based on routine testing data in England, which provide a larger sample size, have not yet been analysed, leaving a gap in the available evidence.

The B.1.1.7 genetic profile includes a deletion of six nucleotides in the S-gene and has been associated with target failures for this gene in polymerase chain reaction (PCR) testing using a three gene assay (ORF1ab, N-gene and S-gene). Although other mutations can also cause an S-gene target failure (SGTF), more than 90% of sequenced SGTF samples since the week of 23 November 2020 were confirmed as matching the B.1.1.7 profile.^2^ Therefore, SGTF provides an indicator from routine PCR testing that can be used as a proxy for B.1.1.7 and that is more rapidly and widely available than sequencing results.

The aim of this study was to assess whether a causal relation exists between infection with the B.1.1.7 variant, compared with infection with wild-type SARS-CoV-2 variants, and the risk of hospital admission. A secondary aim was to re-estimate the mortality risk for patients with the B.1.1.7 variant compared with wild-type variants that has been reported in previous analyses of the study dataset.^6^ ^7^

## Methods

### Identification of patients with confirmed covid-19 by SGTF status

Most PCR tests for SARS-CoV-2 in England are done through the national mass testing programmes.^14^ The pillar 1 testing programme includes testing by hospital and public health laboratories on request of a health professional for a clinical indication, some testing for public health investigations, and occupational testing of health professionals. The pillar 2 testing programme includes large scale PCR testing of respiratory specimens for SARS-CoV-2 infection in Lighthouse laboratories, predominantly for community originated testing.^15^ These laboratories may receive specimens from testing nationwide depending on demand, so individual laboratories do not have a fixed geographical coverage. We identified patients with confirmed covid-19 with SGTF variants from results uploaded to the Second Generation Surveillance System from the three Lighthouse laboratories using TaqPath assays (Milton Keynes, Alderley Park, and Glasgow Lighthouse Laboratories). The identification of these records relied on cycle threshold values being reported into the system from these three laboratories.

We included patients with a positive PCR test for SARS-CoV-2 from pillar 2 between 23 November 2020 and 31 January 2021 and whose specimen had been analysed in one of the TaqPath assay Lighthouse laboratories. Tests from pillar 1 were not analysed at Lighthouse laboratories and hence have not routinely been assessed for SGTF status on a national basis. We defined patients with SGTF variants as those who had cycle threshold values that met the definition for SGTF (ORF1ab and N-gene targets with cycle threshold values ≤30 and no values detected for the S-gene). We defined patients without SGTF variants as those who had cycle threshold values ≤30 at all targets (ORF1ab, N-gene, and S-gene). We chose the inclusion period because SGTF is non-specific to the B.1.1.7 variant and therefore has a low positive predictive value when the prevalence of B.1.1.7 is low. Owing to the increasing prevalence of B.1.1.7, the positive predictive value of SGTF analysis has been >90% for samples collected in England since the week of 23 November 2020.^2^ We stopped the inclusion by 31 January 2021, because >95% of the analysed samples had SGTF variants thereafter.^2^

We extracted laboratory data for all included patients from the Second Generation Surveillance System and included information on the potential confounders age, sex, ethnicity, area of residence, and index of multiple deprivation. The dataset was deduplicated to include each patient’s first positive SARS-CoV-2 test only.

### Assessment of hospital admission and death

We linked all patients to the Secondary Uses Service dataset and the Emergency Care Data Set to obtain information on hospital admissions,^16^ ^17^ as previously described.^18^ The Secondary Uses Service is an administrative dataset that includes healthcare and hospital admission data for completed admissions and treatments submitted to NHS Digital. Secondary Uses Service data are not reported until a hospital admission episode is complete (that is, transfer, discharge, or death); ongoing hospital admissions are not included in this dataset. This information can be complemented with the Emergency Care Data Set, a similar administrative dataset recording attendances at emergency departments, including hospital admissions after emergency department attendance, thus providing another route to capture hospital admission earlier than in the Secondary Uses Service.

We extracted data from the Secondary Uses Service/Emergency Care Data Set and linked them with the laboratory data, including hospital admission records up to 19 May 2021. We classified patients with covid-19 detected in the Secondary Uses Service as being admitted to hospital with covid-19 if they entered the hospital between one and 14 days after their specimen date. If patients were detected in the Emergency Care Data Set only, we classified them as admitted to hospital if they had a discharge status of “admitted” or “transferred” and their attendance date was between one and 14 days after their specimen date. The timeframe of one to 14 days was based on a preliminary descriptive analysis ignoring SGTF status, which indicated that most hospital admissions occurred within 14 days; we explored including later hospital admissions in an additional analysis. Data on the reason for hospital admission were not consistently available, so we included all admissions recorded within this timeframe. We excluded patients who first tested positive on or after their hospital admission to avoid bias of healthcare acquired SARS-CoV-2 infections or testing at admission for non-covid-19 related hospital admission for infection control purposes. Similarly, we excluded patients in hospital within six weeks before testing positive from analysis, because of the possibility of hospital acquired infection.

We further linked the data to a dataset of deaths with covid-19 collated by Public Health England from the following streams: deaths occurring in hospitals and notified to NHS England by NHS trusts, deaths among people testing positive for SARS-CoV-2 notified to Public Health England health protection teams during outbreak management, reports of laboratory test results linked with death reports from NHS records, and death registrations for which covid-19 was mentioned on the death certificate that could be retrospectively linked to a laboratory confirmed SARS-CoV-2 test.^19^

### Potential confounders

The risk of admission to hospital with covid-19 in England has been reported to be positively associated with age, male sex, deprivation, and black or Asian ethnicity.^20^ A previous analysis noted that the prevalence of SGTF variants among patients with covid-19 in England was higher in younger than older age groups.^7^ The B.1.1.7 variant was discovered in southeast England, and the outbreak was initially localised to this and neighbouring regions^4^ ^5^; hence, the prevalence of the B.1.1.7 variant varied by region and calendar period. We therefore treated age, sex, deprivation, ethnicity, region of residence, and date of specimen as potential confounders owing to their known associations with the exposure, outcome, or both.

### Statistical analysis

#### Hospital admission

The primary analysis was a stratified cohort analysis with the aim of estimating the hazard ratio of hospital admission within one to 14 days for patients who tested positive with SGTF compared with non-SGTF variants, while adjusting for confounding. For this outcome, we followed patients from the date of their first positive test until the date of hospital admission if within 14 days or censored them at the date of death or 14 days after the date of first positive test, whichever occurred first. We estimated age group specific absolute risks of hospital admission for patients with SGTF and non-SGTF variants on the basis of models stratified by age group.

Using Cox regression, we estimated the crude hazard ratio of hospital admission within one to 14 days after testing positive for SARS-CoV-2 for patients with SGTF compared with non-SGTF variants. We then estimated an adjusted hazard ratio based on stratification by groups defined by intersecting the potential confounders: 10 year age group, sex, ethnicity, index of multiple deprivation fifth, region of residence (Public Health England Centres), and week of specimen. This model (henceforth referred to as the “base model”) included SGTF status as a binary covariate and additionally included strata specific linear terms for the quantitative covariates age, index of multiple deprivation rank, and calendar date to account for residual confounding from these covariates within each stratum.

We used Schoenfeld tests to test for deviation from the proportional hazard assumption, and we visually assessed the assumption by examining log-log transformed Kaplan-Meier plots for SGTF status and each potential confounder. Because stratification may result in loss of observations, we assessed the effect on the hazard ratio estimate by omitting each of the potential confounders from the stratification set one by one.

Next, we assessed whether the hazard ratio for SGTF was modified by the potential confounders. This was based on likelihood ratio tests between the base model in which the effect of SGTF was assumed constant and the corresponding models that additionally included interaction terms between SGTF status and each stratification covariate.

In additional analyses, we refitted the base model, stratified by lower tier local authority (316 areas) of residence instead of Public Health England Centre (nine regions), and assessed the effect on the results by refitting the base model considering hospital admissions within one to 60 days. For the latter analysis, we allowed for time variation in the hazard ratio for SGTF by fitting a model that assumed piecewise constant hazard ratios by week since positive test and tested for time variation by using a likelihood ratio test of this model compared with the model with constant hazard ratios.

To estimate adjusted absolute risks of hospital admission within 14 days by SGTF status, we fitted a Cox regression model stratified by age group and SGTF status (henceforth referred to as the “absolute risk model”), including main effects for the remaining potential confounders. This model allows estimation of absolute risks, under the assumption of multiplicative effects of each covariate on the hazard. We estimated age group specific absolute risks by SGTF status, evaluated at the mean value of the other potential confounders over all patients. To estimate the corresponding overall absolute risk, we averaged the age group specific estimates over all patients by SGTF status. We used bootstrapping (1000 samples) to estimate 95% confidence intervals for the absolute risks.

#### Mortality

In a secondary analysis, we aimed to estimate the adjusted hazard ratio of death within 28 days of a positive test for patients with SGTF compared with non-SGTF variants. For this, we followed patients from the date of their first positive test until the date of death if deceased within 28 days or otherwise censored them after 28 days. Because the outcome was rarer compared with hospital admission, we estimated the hazard ratio on the basis of Cox regression stratified by age, region of residence, and week of specimen and including main effects for the other potential confounders. We used Stata software (release 14.1) for the statistical analysis.

### Patient and public involvement

This study was observational and based on data from routine healthcare records. No patients were directly involved in the study.

## Results

### Description of patients with covid-19 by SGTF status

During the study period, 839 278 patients with confirmed SARS-CoV-2 infection and valid SGTF status were reported from TaqPath assay Lighthouse laboratories and had not been admitted to hospital within six weeks before testing positive: 592 409 patients with SGTF variants and 246 869 patients with non-SGTF variants. These patients represented 41.0% of all confirmed cases during that time. Table 1 shows the characteristics of the patients with and without SGTF variants. The mean age of patients with SGTF variants was 37.6 years, and the mean age in the non-SGTF group was 37.8 years. Marked differences by region existed, with higher proportions of patients with SGTF variants in London, the East of England, and the South East, as well as differences over time, with most cases of SGTF variants occurring towards the end of December 2020 and the start of 2021, whereas cases without SGTF variants decreased over time.

**Table 1:** Characteristics of patients with and without S-gene target failure (SGTF) variants. Values are numbers (percentages)

Characteristic	Overall (n=839 278)	SGTF (n=592 409)	Non-SGTF (n=246 869)
Age group, years:			
<10	44 216 (5.3)	31 935 (5.4)	12 281 (5.0)
10-19	93 730 (11.2)	63 084 (10.6)	30 646 (12.4)
20-29	160 857 (19.2)	115 296 (19.5)	45 561 (18.5)
30-39	165 570 (19.7)	118 229 (20.0)	47 341 (19.2)
40-49	144 265 (17.2)	102 684 (17.3)	41 581 (16.8)
50-59	132 211 (15.8)	93 468 (15.8)	38 743 (15.7)
60-69	63 897 (7.6)	44 709 (7.5)	19 188 (7.8)
70-79	23 203 (2.8)	15 726 (2.7)	7477 (3.0)
≥80	11 329 (1.3)	7278 (1.2)	4051 (1.6)
Female sex	436 049 (52.0)	305 230 (51.5)	130 819 (53.0)
Region of residence (PHEC):			
East Midlands	44 407 (5.3)	22 913 (3.9)	21 494 (8.7)
East of England	84 454 (10.1)	71 250 (12.0)	13 204 (5.3)
London	169 606 (20.2)	141 864 (23.9)	27 742 (11.2)
North East	48 227 (5.7)	28 120 (4.7)	20 107 (8.1)
North West	151 897 (18.1)	94 050 (15.9)	57 847 (23.4)
South East	128 844 (15.4)	109 794 (18.5)	19 050 (7.7)
South West	26 382 (3.1)	17 235 (2.9)	9147 (3.7)
West Midlands	117 577 (14.0)	74 730 (12.6)	42 847 (17.4)
Yorkshire and Humber	67 884 (8.1)	32 453 (5.5)	35 431 (14.4)
Ethnicity:			
White	615 523 (73.3)	430 930 (72.7)	184 593 (74.8)
Asian	124 156 (14.8)	85 829 (14.5)	38 327 (15.5)
Black	36 778 (4.4)	28 604 (4.8)	8174 (3.3)
Mixed	17 880 (2.1)	13 330 (2.3)	4550 (1.8)
Other	31 491 (3.8)	23 816 (4.0)	7675 (3.1)
Unknown	13 450 (1.6)	9900 (1.7)	3550 (1.4)
Symptoms present	717 627 (85.5)	503 826 (85.0)	213 801 (86.6)
Index of multiple deprivation fifth:			
1 (most deprived)	202 957 (24.2)	132 643 (22.4)	70 314 (28.5)
2	190 807 (22.7)	136 868 (23.1)	53 939 (21.8)
3	162 121 (19.3)	117 727 (19.9)	44 394 (18.0)
4	148 798 (17.7)	106 589 (18.0)	42 209 (17.1)
5 (least deprived)	134 595 (16.0)	98 582 (16.6)	36 013 (14.6)
Specimen collection date:			
23-29 Nov 2020	45 122 (5.4)	7327 (1.2)	37 795 (15.3)
30 Nov-6 Dec 2020	46 205 (5.5)	13 143 (2.2)	33 062 (13.4)
7-13 Dec 2020	65 523 (7.8)	30 817 (5.2)	34 706 (14.1)
14-20 Dec 2020	82 854 (9.9)	52 214 (8.8)	30 640 (12.4)
21-27 Dec 2020	93 442 (11.1)	66 222 (11.2)	27 220 (11.0)
28 Dec 2020-3 Jan 2021	135 516 (16.1)	103 311 (17.4)	32 205 (13.0)
4-10 Jan 2021	132 201 (15.8)	107 269 (18.1)	24 932 (10.1)
11-17 Jan 2021	103 930 (12.4)	89 329 (15.1)	14 601 (5.9)
18-24 Jan 2021	76 521 (9.1)	68 854 (11.6)	7667 (3.1)
25-31 Jan 2021	57 964 (6.9)	53 923 (9.1)	4041 (1.6)

PHEC=Public Health England Centres.

### Hospital admission

Among the 592 409 patients with SGTF variants, 27 710 (4.7%) hospital admissions occurred within one to 14 days, compared with 8523 (3.5%) among the 246 869 patients without SGTF variants. Only 911 (0.15%) patients with SGTF variants and 399 (0.16%) of those without SGTF variants died within 14 days without previous hospital admission; hence, how deaths were treated would make little difference to the hazard ratio estimates for hospital admission.

#### Hazard ratios

The crude hazard ratio of hospital admission within one to 14 days was 1.36 (95% confidence interval 1.33 to 1.40) for patients with versus without SGTF variants. On the basis of the base model, the hazard ratio of hospital admission within one to 14 days was 1.52 (1.47 to 1.57). The proportional hazards assumption was violated for this model (P<0.001). However, this may have reflected a high power to detect minor deviations from proportionality owing to the large sample size, and the corresponding log-log plots showed approximately parallel curves (supplementary figure A).

Of the patients admitted to hospital, 35 769 (98.7%) were included in the analysis; the remaining 464 (1.3%) patients were in single individual strata and therefore uninformative. Of the patients not admitted to hospital, 183 491 (22.8%) were uninformative owing to being in a stratum in which no patients were admitted to hospital. Hence, a total of 655 323 (78.1%) patients were informative for the base model. Removing variables from the stratification set allowed the use of more observations and gave similar results, with hazard ratios ranging from 1.40 to 1.51 (supplementary table A).

Models including interactions between covariates and SGTF status indicated no effect modification by sex (P=0.64), ethnicity (P=0.43), index of multiple deprivation (P=0.69), region of residence (P=0.21), or week (P=0.76). We found evidence that the effect was modified by age (P<0.001), with little difference in hospital admission by SGTF status in patients under 20 but rising to hazard ratios in the range 1.45 to 1.65 in those aged 30 and older (table 2; supplementary table B).

**Table 2:** Hazard ratios of hospital admission within 1-14 days for patients with S-gene target failure (SGTF) variants compared with those without SGTF variants

SGTF status by age group	No (%) admitted to hospital	Stratified base model*—hazard ratio (95% CI): SGTF *v* non-SGTF	Absolute risk model†	
			Hazard ratio (95% CI): SGTF *v* non-SGTF	Absolute risk of 14 day hospital admission—% (95% CI)
**Base model**				
Overall:				
SGTF	27 710/592 409 (4.7)	1.52 (1.47 to 1.57)	1.51 (1.47 to 1.55)	4.7 (4.6 to 4.7)
Non-SGTF	8523/246 869 (3.5)	1.00 (reference)	1.00 (reference)	3.5 (3.4 to 3.5)
**Age group specific model**				
<10 years:				
SGTF	288/31 935 (0.9)	0.93 (0.70 to 1.25)	0.97 (0.79 to 1.20)	0.9 (0.8 to 1.0)
Non-SGTF	121/12 281 (1.0)	1.00 (reference)	1.00 (reference)	1.0 (0.8 to 1.2)
10-19 years:				
SGTF	472/63 084 (0.7)	1.21 (0.99 to 1.49)	1.18 (1.00 to 1.39)	0.7 (0.7 to 0.8)
Non-SGTF	209/30 646 (0.7)	1.00 (reference)	1.00 (reference)	0.7 (0.6 to 0.8)
20-29 years:				
SGTF	2149/115 296 (1.9)	1.29 (1.16 to 1.43)	1.30 (1.19 to 1.42)	1.9 (1.8 to 1.9)
Non-SGTF	707/45 561 (1.6)	1.00 (reference)	1.00 (reference)	1.5 (1.4 to 1.7)
30-39 years:				
SGTF	3964/118 229 (3.4)	1.45 (1.34 to 1.58)	1.41 (1.32 to 1.51)	3.4 (3.3 to 3.5)
Non-SGTF	1216/47 341 (2.6)	1.00 (reference)	1.00 (reference)	2.6 (2.4 to 2.7)
40-49 years:				
SGTF	5162/102 684 (5.0)	1.61 (1.50 to 1.74)	1.59 (1.50 to 1.69)	5.0 (4.9 to 5.2)
Non-SGTF	1429/41 581 (3.4)	1.00 (reference)	1.00 (reference)	3.4 (3.3 to 3.6)
50-59 years:				
SGTF	6734/93 468 (7.2)	1.58 (1.48 to 1.69)	1.58 (1.50 to 1.67)	7.2 (7.0 to 7.4)
Non-SGTF	1902/38 743 (4.9)	1.00 (reference)	1.00 (reference)	4.9 (4.7 to 5.1)
60-69 years:				
SGTF	4733/44 709 (10.6)	1.65 (1.53 to 1.79)	1.63 (1.53 to 1.73)	10.6 (10.3 to 10.9)
Non-SGTF	1361/19 188 (7.1)	1.00 (reference)	1.00 (reference)	7.1 (6.8 to 7.5)
70-79 years:				
SGTF	2653/15 726 (16.9)	1.45 (1.32 to 1.60)	1.49 (1.38 to 1.60)	16.9 (16.3 to 17.5)
Non-SGTF	932/7477 (12.5)	1.00 (reference)	1.00 (reference)	12.5 (11.7 to 13.2)
≥80 years:				
SGTF	1555/7278 (21.4)	1.60 (1.41 to 1.82)	1.50 (1.36 to 1.64)	21.7 (20.7 to 22.7)
Non-SGTF	646/4051 (15.9)	1.00 (reference)	1.00 (reference)	16.2 (15.0 to 17.3)

* Hazard ratios from primary stratified models estimated on basis of Cox regression stratified by potential confounders 10 year age group, sex, ethnicity, index of multiple deprivation (IMD) fifth, region of residence, and week of specimen collection and using regression adjustment for quantitative covariates age, IMD rank, and date of specimen.

† Secondary absolute risk model based on Cox regression stratified by SGTF status and age group only and regressed on remaining potential confounders. 14 day absolute hospital admission risks by SGTF status estimated on basis of absolute risk model, at mean levels of potential confounders.

When we refitted the base model using a finer geographic stratification (lower tier local authority) for the areas of residence instead of Public Health England Centres, 29 481 (81.4%) of the hospital admissions and 204 469 (24.4%) of the observations could be used. The adjusted hazard ratio was 1.52 (1.47 to 1.58).

Extending the follow-up time, 46 371 (7.8%) patients with SGTF variants and 16 654 (6.7%) without SGTF variants had been admitted to hospital within one to 60 days of the first positive test. The crude hazard ratio of hospital admission within one to 60 days was 1.17 (1.15 to 1.19), and the corresponding fully stratified hazard ratio was 1.25 (1.22 to 1.28). The proportional hazards assumption was violated (P<0.001). Consistently, the hazard ratio for SGTF varied with time since first positive test when we allowed for a time varying effect (P<0.001). The estimated hazard ratios were 1.46 (1.40 to 1.52) in days one to seven after specimen collection and 1.62 (1.54 to 1.70) in days eight to 14, but they were subsequently close to 1.0 (range 0.91 to 1.03; supplementary table C).

#### Absolute risks

**Fig 1:**
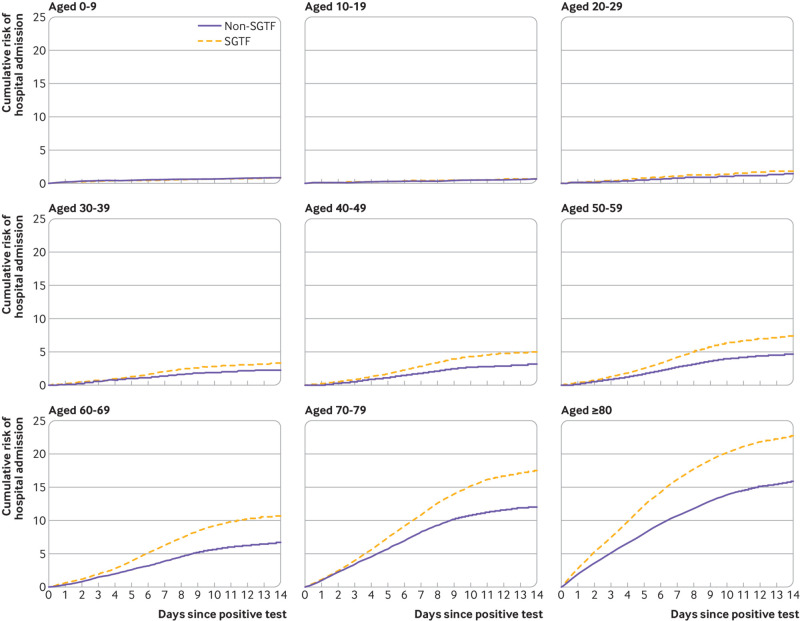
Cumulative risk of hospital admission within 1-14 days after positive SARS-CoV-2 test, by age group. Risks were estimated with Cox regression stratified by S-gene target failure (SGTF) status and age group, adjusted for sex, index of multiple deprivation fifth, ethnicity, region of residence, and calendar week (potential confounders set to mean covariate levels)

Table 2 shows the hazard ratio estimates for SGTF from the absolute risk model, which were similar to those from the fully stratified base model, both overall and by age group. On the basis of this model, table 2 and fig 1 show estimates of the age group specific absolute risks of hospital admission within 14 days after first positive test by SGTF status, at average levels of the potential confounders over all patients. The overall estimated absolute risk of hospital admission after 14 days was 4.7% (95% confidence interval 4.6% to 4.7%) for patients with SGTF variants and 3.5% (3.4% to 3.5%) for those without SGTF variants. Most of the hospital admissions were in the first 14 days; supplementary figure B shows the corresponding graphs to 60 days, which show a high hospital admission rate over the first 14 days and an approximately constant low rate subsequently.

### Mortality

A total of 2603 (0.44%) deaths occurred within 28 days of positive test in the 592 409 patients with SGTF variants and 899 (0.36%) deaths within 28 days in the 246 869 patients without SGTF variants. The crude hazard ratio of death was 1.22 (1.13 to 1.31). After adjustment for all considered potential confounders, the hazard ratio was 1.59 (1.44 to 1.74).

## Discussion

This retrospective analysis of patients with covid-19 identified through community testing in England indicated that the risk of hospital admission within 14 days after a positive test was 1.52 (1.47 to 1.57) times higher for patients diagnosed with covid-19 and infected with the B.1.1.7 variant compared with those infected with wild-type variants, after adjustment for age, sex, deprivation, ethnicity, region, and week of diagnosis. Consistent with previously reported analyses of the study dataset,^6^ ^7^ the results indicated that B.1.1.7 is associated with a 1.59 (1.44 to 1.74) times higher risk of death within 28 days than wild-type variants.

The results indicated that the higher risk of hospital admission may apply primarily to adults above the age of 30, and the risk was not found to be higher for patients with versus without SGTF variants below the age of 20. This may reflect a similarly low risk of severe disease in younger and less comorbid people, as previously reported for wild-type variants.^20^

We have provided absolute risk estimates of hospital admission within 14 days by age group after adjustment for potential confounders. Under the model used to estimate the absolute risks, the B.1.1.7 variant can be estimated to have caused an excess 8941 hospital admissions within 14 days during the study period, on the basis of a comparison of the observed hospital admissions with the expected hospital admissions under a counterfactual scenario in which patients with SGTF variants were admitted to hospital at the same rate as those with non-SGTF variants. However, this estimate ignores the excess transmissibility of B.1.1.7,^2^ ^4^ ^5^ and hence it likely underestimates the total number of hospital admissions attributable to B.1.1.7.

### Strengths and limitations of study

Strengths of this analysis include the use of a community based dataset, which includes all patients with covid-19 identified through community testing in England. The large dataset allowed for the use of stratification to adjust for confounding due to individual level demographic and socioeconomic factors, region, and calendar period.

Although the observed increased risks of hospital admission and mortality are both consistent with the hypothesis of increased severity of B.1.1.7 compared with wild-type SARS-CoV-2, hospital admission may not be susceptible to the same confounding pathways as mortality. The calendar period in which the prevalence of the B.1.1.7 increased coincided with a general increase in diagnoses of covid-19 in the UK,^21^ and initial outbreaks of B.1.1.7 were local to southeast England and neighbouring regions.^5^ High local pressure on the healthcare system might lead to higher mortality because of factors such as insufficient resources and staff per patient admitted to hospital. Hence, because the pressure on the healthcare system was higher in areas where B.1.1.7 was more prevalent, those areas may have seen a somewhat higher mortality due to hospital over-burden. This could result in positive confounding—that is, the mortality risk associated with B.1.1.7 may have been somewhat overestimated in this and previous analyses of community testing data in England.^6^ ^7^ This and previous studies did not directly adjust for hospital pressure, and the potential confounding effect of local hospital pressure was accounted for only indirectly through adjustment for time period and region.^6^ ^7^ ^8^ By contrast, high local hospital pressure is not associated with the propensity that patients newly diagnosed as having covid-19 experience disease sufficiently severe to require admission to hospital and is unlikely to lead to a greater proportion of patients with covid-19 needing hospital care being admitted to hospital. Hospital pressure is thus unlikely to positively confound the association between SGTF status and hospital admission. Most likely, hospital pressure does not affect the propensity that a patient severely affected with covid-19 is admitted to hospital. If so, hospital pressure does not confound the association between SGTF status and hospital admission. However, owing to severe local hospital over-burden, some patients severely affected by covid-19 who would otherwise have been admitted to hospital may not have been admitted or were admitted later than they otherwise would. Because of the time and region specific overlap between high hospital pressure and high prevalence of the B.1.1.7 variant, this would, however, be expected to result in negative confounding and hence underestimation of the hazard ratio of hospital admission for B.1.1.7 infected patients. In the absence of full control for the potential confounding due to local hospital pressure in analyses of mortality risk, the association with hospital admission corroborates the hypothesis that the B.1.1.7 variant is associated with more severe disease than wild-type variants.

Our study also has limitations. The study population included patients with covid-19 with known SGTF status from three laboratories providing community testing. These laboratories do a large proportion of tests from across the country (41.0% of positive cases over the study period), but geographic distribution can vary depending on capacity from other available laboratories. The adjusted model accounted for these potential geographical differences in testing coverage. The community based population excluded patients whose covid-19 was diagnosed after they presented directly to emergency or other healthcare services. Patients who present directly to healthcare services may have more severe disease than those whose diagnosis results from community testing. Community testing is largely self-selected, and we cannot control for the possibility that testing patterns may have differed between patients infected with the B.1.1.7 variant and patients infected with wild-type variants. Some evidence suggests that people infected with the B.1.1.7 variant are more likely to experience symptomatic disease compared with other infected patients,^22^ but whether the B.1.1.7 variant is also associated with more severe symptoms in the subset who experience symptoms is unknown.

Our analysis is limited by a lack of data on comorbidity and obesity, which are risk factors for hospital admission with covid-19.^20^ Previous studies have, however, not noted any association between B.1.1.7 status and body mass index or comorbidity in patients with covid-19.^8^ ^9^ Hence, we do not expect that these potential confounders were strongly associated with SGTF status. Furthermore, they were accounted for indirectly through age, sex, ethnicity, and deprivation. In light of the finding that the risk of hospital admission for patients infected with the B.1.1.7 variant increased with age, further research is needed to understand whether the severity associated with the B.1.1.7 variant is modified by age and associated factors such as comorbidity and obesity.

The analysis uses SGTF status, which is a proxy test for the B.1.1.7 variant. However, any non-differential misclassification is likely to result in a small bias towards the null, and the available sequencing data indicate that the positive and negative predictive values of the SGTF test were >90% during the studied period.^2^

Healthcare recommendations for patients with covid-19 did not differ by SARS-CoV-2 variant, but the B.1.1.7 variant and the reports of its increased severity saw great media attention over the studied period, and this might have affected the healthcare seeking behaviour of patients with covid-19. However, the assessment of SGTF was done for surveillance purposes, and the SGTF status was not routinely provided to patients or their healthcare providers, so we have no reason to believe that healthcare seeking behaviour differed by SGTF status. Consistently, we observed no significant variation in the hazard ratio for patients with or without SGTF variants by calendar week.

Delays in reporting might affect this analysis, particularly for the hospital admission data from the Secondary Uses Service, which are not recorded until after the completion of a hospital episode. However, at least 108 days had elapsed between the patients’ dates of positive test and the linkage with the hospital admission data, which means delays likely had limited effect. Furthermore, the patients with SGTF variants were on average found to have covid-19 later in calendar time than those without SGTF variants. Hence, any non-differential under-reporting of hospital admissions due to reporting delays may have resulted in underestimation of the risk of hospital admission predominantly in the patients with SGTF variants, which might have resulted in a small underestimation of the true hazard ratio. Our analyses controlled for calendar week, which likely limits the effect of this. Additionally, the use of hospital admission data from health service datasets that principally serve administrative purposes has the benefit of broad coverage.

The proportional hazards assumption of the Cox regression model was violated, but the corresponding log-log plots indicated that the deviation from proportionality was very minor within the first 14 days after the positive test. Consistently, when we allowed for time dependent effects, the time varying hazard ratios in the first 14 days were similar to the overall hazard ratio of hospital admission within one to 14 days from the primary analysis. By contrast, the time dependent hazard ratios of hospital admission 15-60 days after the positive test from the additional analysis in which we extended the follow-up time beyond 14 days were close to 1.0, and the hazards were hence clearly non-proportional over this extended follow-up. These patterns likely reflect the fact that the hospital admission data were limited by a lack of information on the reason for admission and the analysis was therefore based on hospital admissions due to any cause. Because of the narrow time interval considered in the primary analysis of hospital admissions between one and 14 days after a first positive SARS-CoV-2 test, most of these admissions were likely due to covid-19. Assuming that the background rate of hospital admission due to non-covid-19 causes was similar between patients with and without SGTF variants, the resulting non-differential misclassification of hospital admissions due to other causes may, however, have resulted in a slight underestimation of the cause specific hazard ratio in days one to 14. Such misclassification is a likely explanation for the attenuation of the hazard ratio when we extended the follow-up period to 60 days, because non-covid-19 causes may constitute a higher proportion of the reasons for the late hospital admissions.

### Comparison with other studies

The estimated higher risk of hospital admission is in line with a previous analysis that estimated a hazard ratio of hospital admission of 1.34 (1.07 to 1.66), based on follow-up of patients with covid-19 with sequencing confirmed B.1.1.7 or wild-type SARS-CoV-2 in England.^13^ That study observed only 120 hospital admissions in B.1.1.7 infected patients, which yielded a wide confidence interval. By contrast, in this study we observed 27 710 hospital admissions in patients with SGTF variants, which allowed us to provide estimates with higher precision. The results are also consistent with a previous ecological analysis that estimated a relative risk for hospital admission of 1.7 based on hospital admission patterns by the regional prevalence of SGTF variants in England,^11^ ^12^ as well as with reported relative risks in the range 1.6-1.7 based on data from Scotland,^11^ seven EU/EEA countries,^23^ Denmark,^24^ and Canada.^10^ This adds to the evidence of higher severity covid-19 after infection with B.1.1.7 compared with wild-type SARS-CoV-2, as further indicated by its association with higher risk of intensive care admission and death.^6^ ^7^ ^8^ ^9^ ^10^ ^23^

Our finding of lower age specific hazard ratio estimates in younger age groups are consistent with a previously reported age specific adjusted odds ratio of hospital admission of 1.0 for patients aged 0-19 with versus without SGTF variants in seven EU/EEA countries but contrasts with a study in Denmark that reported an adjusted odds ratio of 1.84 for patients aged 0-29 with SGTF variants.^23^ ^24^ Children and adolescents aged ≤18 who were admitted to hospital with covid-19 in November 2020 to January 2021 at King’s College Hospital in London (where the local prevalence of B.1.1.7 was high) were reported to have had similar clinical severity and treatment requirements to those admitted in March to May 2020,^25^ corroborating the suggestion that patients in the youngest age groups experience no more severe disease if infected with B.1.1.7 than with wild-type SARS-CoV-2.

### Conclusions

The results from this large nationwide community testing cohort suggest that the risk of hospital admission is higher for patients with covid-19 infected with the B.1.1.7 variant compared with wild-type variants, likely reflecting association of the variant with more severe disease. This higher severity may, however, be specific to adults older than 30 years, and further research is needed to determine whether the severity is modified by factors associated with ageing. Taken together with the previous evidence of increased mortality and transmissibility, the results suggest that epidemics of the B.1.1.7 variant are likely to result in higher burden on the healthcare system in unvaccinated populations compared with epidemics of wild-type SARS-CoV-2.

**What is already known on this topic**

The SARS-CoV-2 B.1.1.7 variant was discovered in England in December 2020 and thereafter became the dominant lineage owing to a higher transmissibility than wild-type SARS-CoV-2
Some evidence suggests that B.1.1.7 is associated with more severe disease, but the studies that have found an association with increased mortality may have been limited by confounding
Hospital admission as a measurement of disease severity is less likely than mortality to be positively confounded by hospital burden

**What this study adds**

Patients with covid-19 who tested positive for the B.1.1.7 variant had a 1.52-fold hazard of hospital admission within 1-14 days of the first positive test compared with wild-type variants
The results likely reflect a more severe disease associated with the SARS-CoV-2 B.1.1.7 variant, particularly in patients aged 30 or older

## Web extra

Extra material supplied by authors

Web appendix: Supplementary materials

## Notes

Contributors: TN, KAT, RJH, SRS, AC, DDA, GD, and AMP designed the study. KAT, RJH, JF, HA, AC, and GD contributed to the linkage of the data sources. RJH and KAT did the statistical analysis. TN, KAT, RJH, GD, and AMP wrote the manuscript. SRS, JF, HA, AC, and DDA reviewed and revised the manuscript. AMP, AC, and DDA acquired funding. TN is the guarantor. The corresponding author attests that all listed authors meet authorship criteria and that no others meeting the criteria have been omitted.

Ethical approval: This surveillance was done as part of Public Health England’s responsibility to monitor covid-19 during the current pandemic. Public Health England has legal permission, provided by Regulation 3 of The Health Service (Control of Patient Information) Regulations 2002, to process confidential patient information (https://www.legislation.gov.uk/uksi/2002/1438/regulation/3/made) under Sections 3(i) (a) to (c), 3(i)(d) (i) and (ii) and 3(3) as part of its outbreak response activities. As such, this work falls outside the remit for ethical review.

Data sharing: Requests to access non-publicly available data are handled by the Public Health England Office for Data Release (https://www.gov.uk/government/publications/accessing-public-health-england-data/about-the-phe-odr-and-accessing-data/).

The lead author affirms that the manuscript is an honest, accurate, and transparent account of the study being reported; that no important aspects of the study have been omitted; and that any discrepancies from the study as planned (and, if relevant, registered) have been explained.

Dissemination to participants and related patient and public communities: Because the study was observational and involved routine medical records without direct contact with patients, dissemination of the results to study participants is not applicable. We will publish a lay summary for the public on the website of the MRC Biostatistics Unit at the University of Cambridge. We plan to refer to the publication findings in future Public Health England reports that appear on the Public Health England website, for awareness for the public and clinicians. These are accompanied by appropriate media handling to support the media in reporting to the public. We will also disseminate the research findings via Twitter.

Provenance and peer review: Not commissioned; externally peer reviewed.
