# Supplementary material for: Risk of hospital admission for patients with SARS-CoV-2 variant B.1.1.7: cohort analysis
Source: BMJ. 2021 Jun 15;373:n1412. doi: 10.1136/bmj.n1412 (PMC8204098; doi:10.1136/bmj.n1412)
Supplement: Supplementary file 1 — Web appendix: Supplementary materials [file nybt065698.ww1.pdf]

Supplementary material for:

Risk of hospital admission for patients with SARS-CoV-2 variant B.1.1.7: cohort analysis

Tommy Nyberg, *research associate* [a]*
Katherine A. Twohig, *senior epidemiology scientist* [b]
Ross J. Harris, *senior statistician* [c]
Shaun R. Seaman, *senior research associate* [a]
Joe Flannagan, *senior epidemiology scientist* [b]
Hester Allen, *principal epidemiology scientist* [b]
Andre Charlett, *head of department* [c]
Daniela De Angelis, *professor of statistical science for health* [a,c]
Gavin Dabrera, *consultant in public health medicine* [b]
Anne M. Presanis, *senior investigator statistician* [a]

[a] MRC Biostatistics Unit, University of Cambridge, Cambridge, United Kingdom.
[b] COVID-19 National Epidemiology Cell, Public Health England, London, United Kingdom.
[c] National Infection Service, Public Health England, London, United Kingdom.

* Corresponding author.

Supplementary Figure A. Plots of the natural logarithm of time since positive test versus the -log-log transformed Kaplan-Meier estimate of the survival function. If the proportional hazards assumption made by the Cox regression model is true, these lines are expected to be parallel.

(a) By SGTF status.

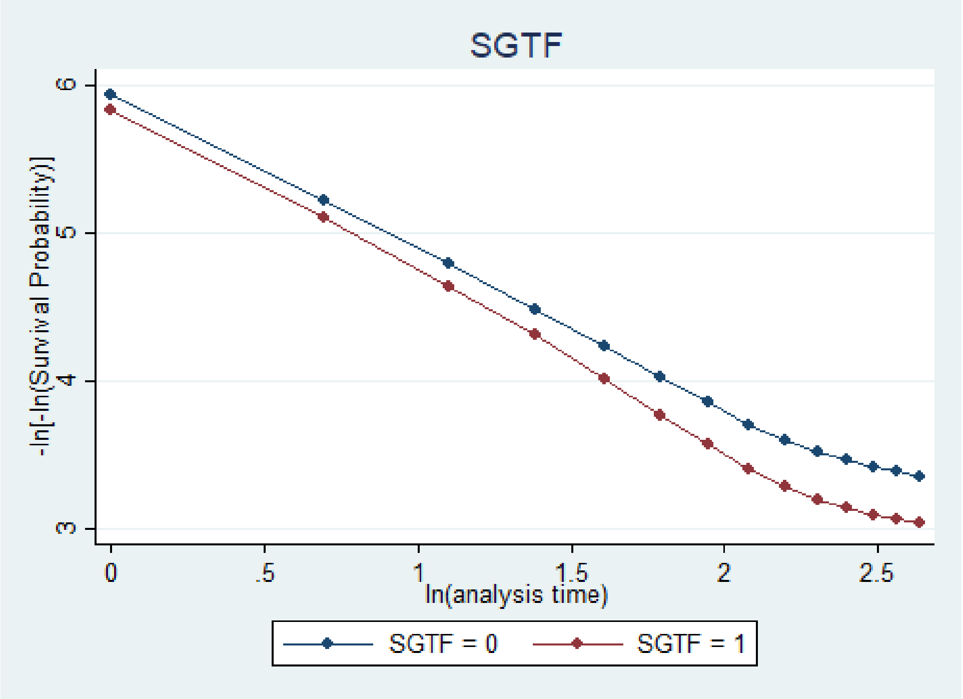

(b) By age group.

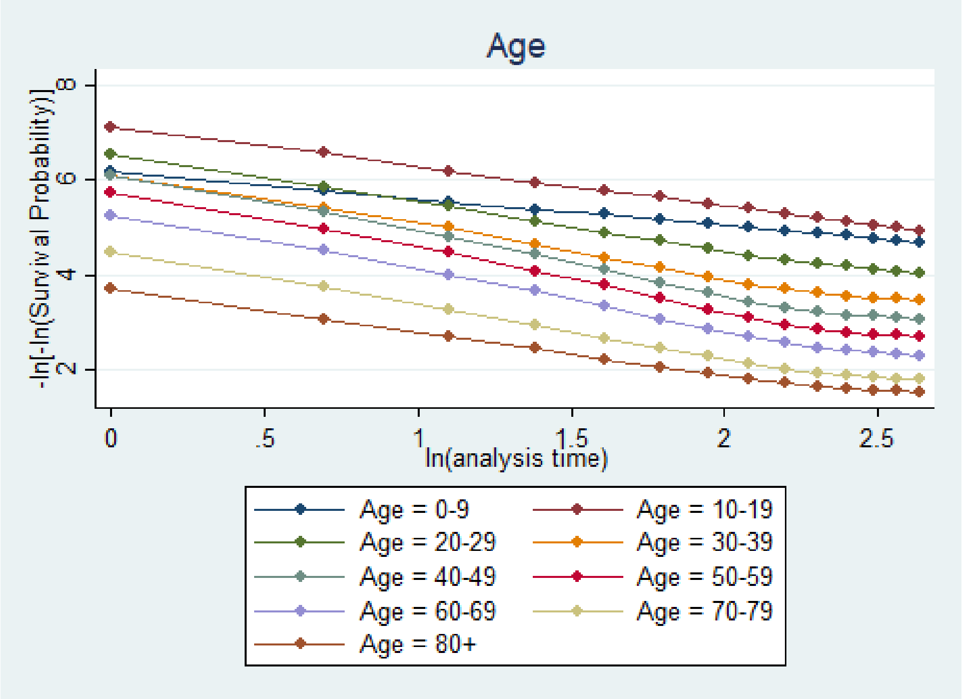

(c) By sex.

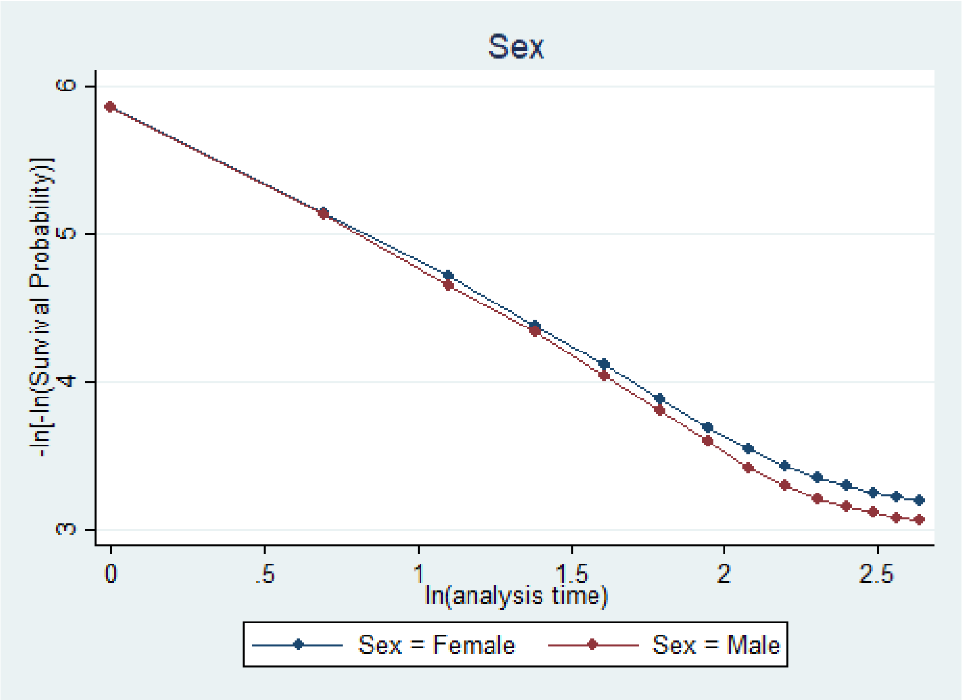

(d) By region of residence (PHEC).

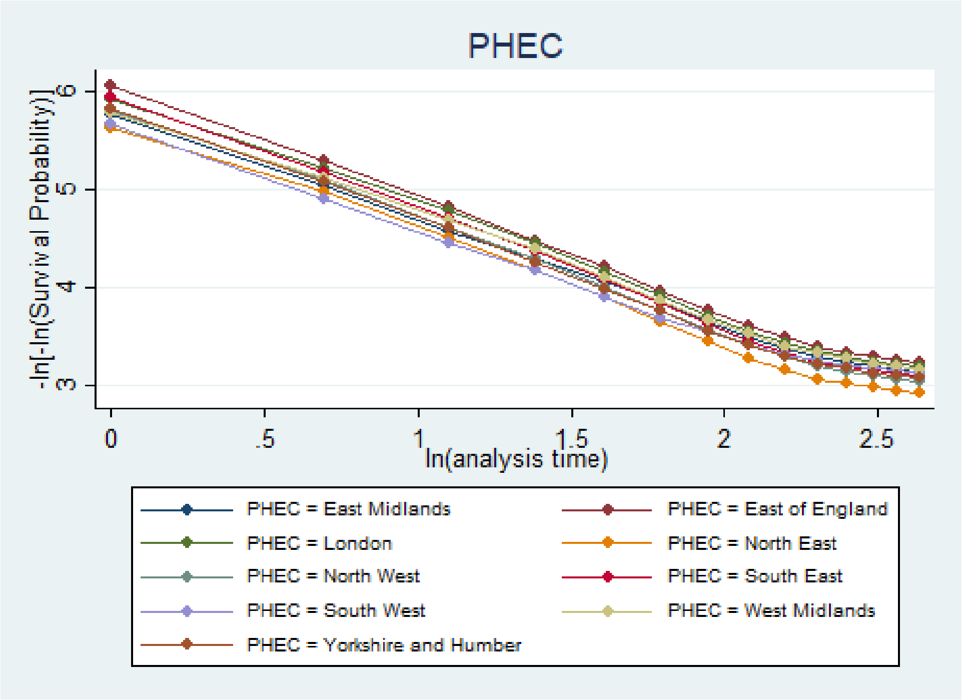

(e) By ethnicity.

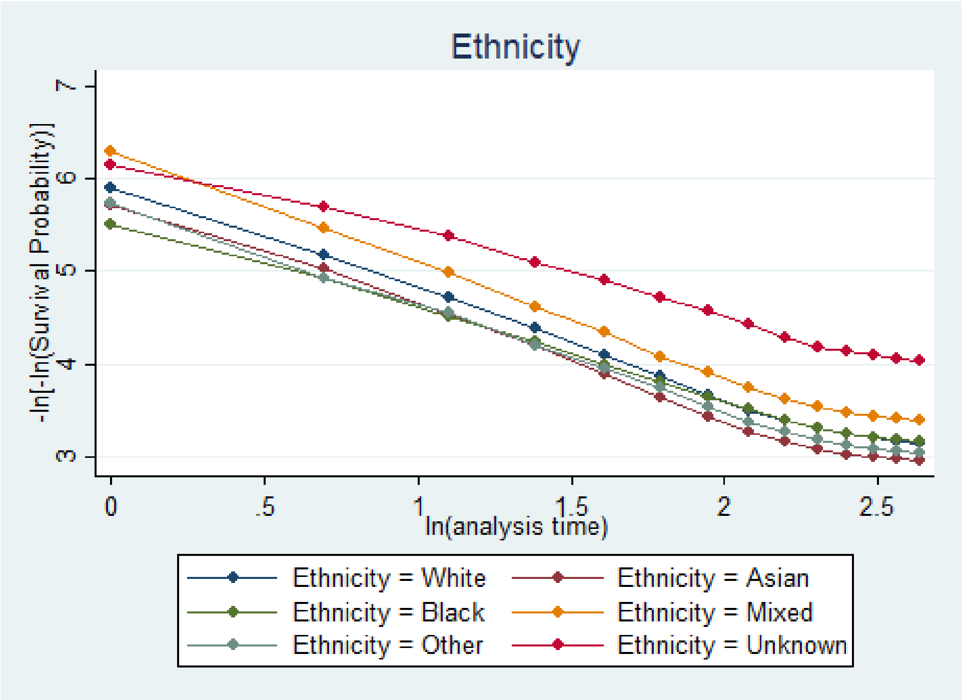

(f) By index of multiple deprivation quintile.

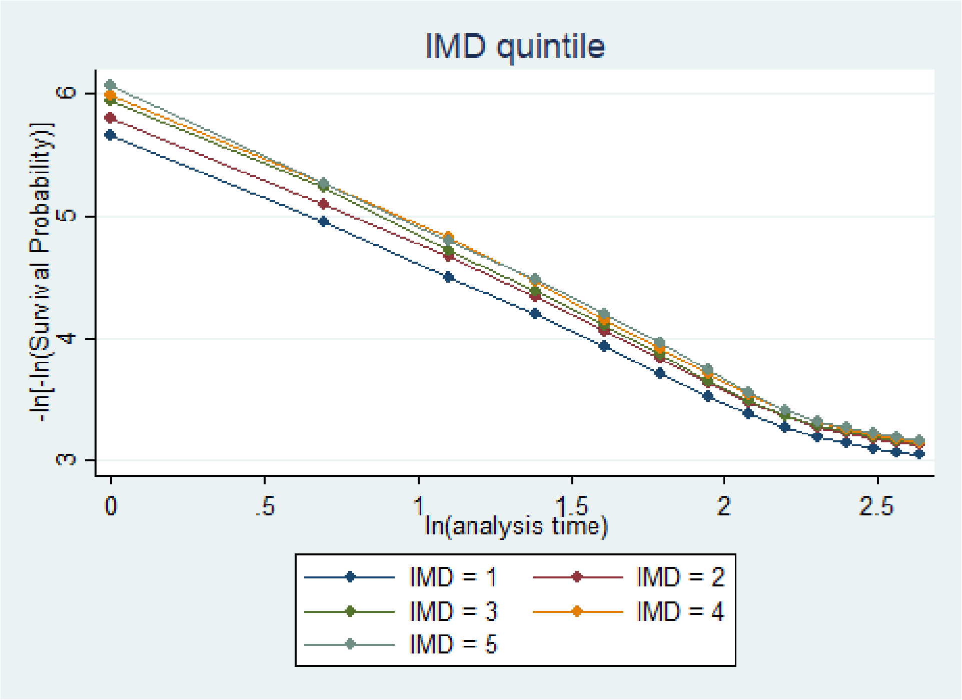

(g) By week of positive test.

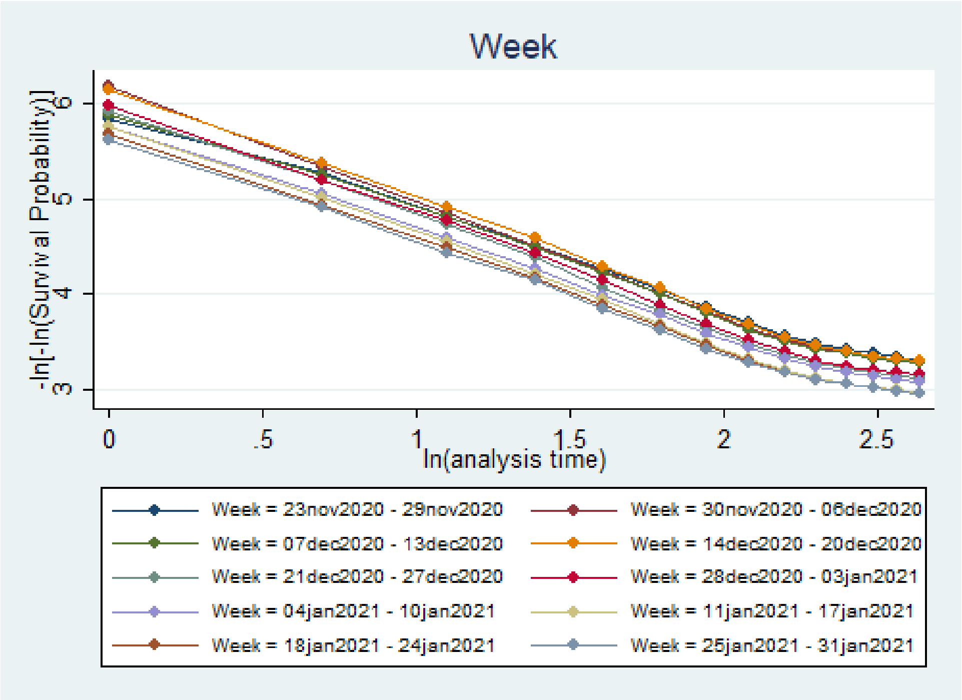

Supplementary Figure B. Cumulative hospitalisation risk within 1-60 days after positive COVID-19 test, by age group. The risks were estimated based on a Cox regression model stratified by SGTF status and age group, adjusted for sex, IMD quintile, ethnicity, region of residence, and calendar week (potential confounders set to mean covariate levels).

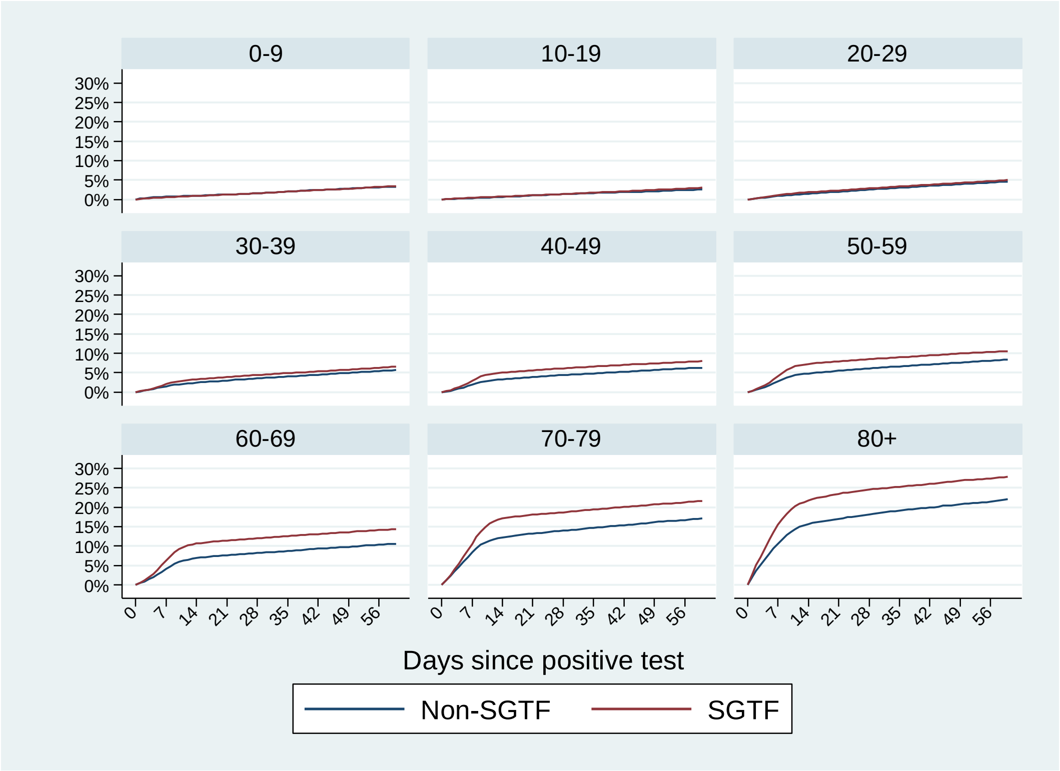

Supplementary Table A. Number of hospitalisations and number of observations that were informative, and the corresponding hazard ratios of hospitalisation within 1-14 days for SGTF compared to non-SGTF patients, for the fully stratified "base model", and for corresponding reduced models with each stratification covariate excluded one-by-one. The hazard ratios were estimated stratified by the adjustment covariates 10-year age group, sex, ethnicity, IMD quintile, region of residence, and/or week of specimen; and using regression adjustment for the quantitative covariates age, IMD rank, and/or date of specimen.

**Stratification variables**	**N hospitalisations included (%)**	**N observations included (%)**	**SGTF HR (95% CI)**
Stratification for all potential confounders: age, sex, ethnicity, IMD, PHEC, week ("base model")	35769 (98.7%)	655323 (78.1%)	1.52 (1.47 to 1.57)
Excluding: age;<br>Stratification for: sex, ethnicity, IMD, PHEC, week	36226 (100.0%)	812466 (96.8%)	1.40 (1.35 to 1.44)
Excluding: sex;<br>Stratification for: age, ethnicity, IMD, PHEC, week	35991 (99.3%)	720843 (85.9%)	1.51 (1.46 to 1.55)
Excluding: PHEC;<br>Stratification for: age, sex, ethnicity, IMD, week	36198 (99.9%)	791640 (94.3%)	1.43 (1.39 to 1.47)
Excluding: ethnicity;<br>Stratification for: age, sex, IMD, PHEC, week	36221 (100.0%)	750350 (89.4%)	1.49 (1.44 to 1.53)
Excluding: IMD;<br>Stratification for: age, sex, ethnicity, PHEC, week	36137 (99.7%)	772225 (92.0%)	1.50 (1.45 to 1.54)
Excluding: week;<br>Stratification for: age, sex, ethnicity, IMD, PHEC	36204 (99.9%)	805192 (95.9%)	1.46 (1.43 to 1.50)

Supplementary Table B. Tests for interaction and hazard ratios of hospitalisation within 1-14 days for SGTF compared to non-SGTF patients by all considered covariates, based on stratified Cox regression. The hazard ratios were estimated stratified by the adjustment covariates 10-year age group, sex, ethnicity, IMD quintile, region of residence, and week of specimen; and using regression adjustment for the quantitative covariates age, IMD rank, and date of specimen.

**Covariate**	**Category**	**HR (95% CI)**
Age group<br>(test for interaction, P<0.001)	<10	0.93 (0.70 to 1.25)
	10-19	1.21 (0.99 to 1.49)
	20-29	1.29 (1.16 to 1.43)
	30-39	1.45 (1.34 to 1.58)
	40-49	1.61 (1.50 to 1.74)
	50-59	1.58 (1.48 to 1.69)
	60-69	1.65 (1.53 to 1.79)
	70-79	1.45 (1.32 to 1.60)
	80+	1.60 (1.41 to 1.82)
Sex<br>(test for interaction, P=0.64)	Female	1.53 (1.47 to 1.60)
	Male	1.51 (1.45 to 1.58)
Region of residence (PHEC)<br>(test for interaction, P=0.21)	East Midlands	1.57 (1.37 to 1.79)
	East of England	1.38 (1.22 to 1.55)
	London	1.50 (1.38 to 1.62)
	North East	1.58 (1.43 to 1.76)
	North West	1.53 (1.44 to 1.63)
	South East	1.61 (1.46 to 1.76)
	South West	1.78 (1.50 to 2.12)
	West Midlands	1.53 (1.41 to 1.65)
	Yorkshire & Humber	1.41 (1.28 to 1.55)
Ethnicity<br>(test for interaction, P=0.43)	White	1.53 (1.48 to 1.59)
	Asian	1.55 (1.43 to 1.67)
	Black	1.39 (1.17 to 1.66)
	Mixed	1.53 (1.13 to 2.08)
	Other	1.39 (1.17 to 1.66)
	Unknown	1.01 (0.62 to 1.64)
Index of multiple deprivation<br>(test for interaction, P=0.69)	Quintile 1 – most deprived	1.53 (1.44 to 1.62)
	Quintile 2	1.47 (1.38 to 1.57)
	Quintile 3	1.51 (1.41 to 1.62)
	Quintile 4	1.53 (1.42 to 1.65)
	Quintile 5 – least deprived	1.59 (1.46 to 1.72)
Week of positive test<br>(test for interaction, P=0.76)	23/11/2020 – 29/11/2020	1.50 (1.29 to 1.74)
	30/11/2020 – 06/12/2020	1.53 (1.34 to 1.74)
	07/12/2020 – 13/12/2020	1.57 (1.42 to 1.75)
	14/12/2020 – 20/12/2020	1.58 (1.44 to 1.74)
	21/12/2020 – 27/12/2020	1.61 (1.48 to 1.76)
	28/12/2020 – 03/01/2021	1.46 (1.36 to 1.57)
	04/01/2021 – 10/01/2021	1.50 (1.39 to 1.62)
	11/01/2021 – 17/01/2021	1.45 (1.32 to 1.58)

	18/01/2021 – 24/01/2021	1.56 (1.38 to 1.77)
	25/01/2021 – 31/01/2021	1.54 (1.30 to 1.82)

Supplementary Table C. Hazard ratios of hospitalisation within 1-60 days for SGTF compared to non-SGTF patients, based on stratified Cox regression allowing for time-varying (piecewise constant) HRs for SGTF status. The hazard ratios were estimated stratified by the adjustment covariates 10-year age group, sex, ethnicity, IMD quintile, region of residence, and week of specimen; and using regression adjustment for the quantitative covariates age, IMD rank, and date of specimen.

**Days after positive test**	**HR (95% CI)**
1-7 days	1.46 (1.40 to 1.52)
8-14 days	1.62 (1.54 to 1.70)
15-21 days	1.03 (0.96 to 1.11)
22-28 days	0.91 (0.84 to 0.99)
29-35 days	0.99 (0.91 to 1.08)
36-42 days	0.97 (0.89 to 1.05)
43-49 days	1.02 (0.94 to 1.12)
50-60 days	0.95 (0.89 to 1.02)
